# Comparison of clinical outcomes following percutaneous coronary intervention versus optimal medical therapy based on gray-zone fractional flow reserve in stable angina patients with intermediate coronary artery stenosis (COMFORTABLE prospective study): Study protocol for a multicenter randomized controlled trial

**DOI:** 10.1186/s13063-019-3182-1

**Published:** 2019-01-28

**Authors:** Hironori Kitabata, Takashi Kubo, Yasutsugu Shiono, Kunihiro Shimamura, Yasushi Ino, Takashi Tanimoto, Yasushi Hayashi, Kenichi Komukai, Hiromichi Sougawa, Keizo Kimura, Masahiro Gohda, Toshikazu Hashizume, Masahiro Obana, Kazuisa Maeda, Junichi Yamaguchi, Takashi Akasaka

**Affiliations:** 1 Department of Cardiovascular Medicine, Wakayama Medical University, 811-1, Kimiidera, Wakayama, Wakayama 641-8509 Japan; 2 Department of Cardiovascular Medicine, Shingu Municipal Medical Center, 18-7 Hachibuse, Shingu, Wakayama, 647-0072 Japan; 3 Department of Cardiovascular Medicine, Wakayama Rosai Hospital, 93-1 Kinomoto, Wakayama, Wakayama 640-8505 Japan; 4 Department of Cardiovascular Medicine, Hidaka General Hospital, 116-2 Sono, Gobo, Wakayama, 644-0002 Japan; 5 Department of Cardiovascular Medicine, Hashimoto Municipal Hospital, 2-8-1Ominedai, Hashimoto, Wakayama, 648-0005 Japan; 6 Department of Cardiovascular Medicine, Kinan Hospital, 46-7 Shinjocho, Tanabe, Wakayama, 646-8588 Japan; 7 Department of Cardiovascular Medicine, Seiyu Memorial Hospital, 391 Nishitai, Wakayama, 649-6335 Japan; 8 Department of Cardiovascular Medicine, Minami Wakayama Medical Center, 27-1 Takinaicho, Tanabe, Wakayama, 646-8558 Japan; 9 Department of Cardiovascular Medicine, Saiseikai Wakayama Hospital, 45 Junibancho, Wakayama, 640-8158 Japan; 10 Department of Cardiovascular Medicine, Naga Municipal Hospital, 1282 Uchita, Kinokawa, 649-6414 Japan; 11 Department of Cardiology, The Heart Institute of Japan, Tokyo Women’s Medical University, 8-1 Kawada-cho, Shinjuku-ku, Tokyo, 162-8666 Japan

**Keywords:** Coronary physiology, Fractional flow reserve, Medical therapy, Percutaneous coronary intervention

## Abstract

**Background:**

Even in the current drug-eluting stent era, revascularization for coronary stenosis with fractional flow reserve (FFR) between 0.75 and 0.80, the so-called “gray zone,” is a matter of debate. Previous studies have reported conflicting results regarding outcomes of revascularization versus deferral for coronary stenosis when FFR values are in the gray zone, but these studies have had differing designs and populations. We therefore will investigate whether medical therapy plus percutaneous coronary intervention (PCI) is superior to medical therapy alone in reducing major cardiovascular events in patients presenting with coronary stenosis with gray zone FFR values.

**Methods/design:**

This is a prospective, multicenter, open-label, parallel group, randomized, controlled, superiority study. A total of 410 eligible participants will be recruited and randomized to either the medical therapy plus PCI group or the medical therapy alone group. The primary endpoint is 1-year major adverse cardiac events (MACEs), defined as a combined endpoint of all-cause death, nonfatal myocardial infarction (MI), or unplanned target vessel revascularization (TVR). Secondary endpoints include MACE at 2 and 5 years. Moreover, each individual component of the primary endpoint, cardiovascular death, target vessel-related and non-target vessel-related MI, all MI, clinically driven TVR or non-TVR, all revascularization, stent thrombosis, and angina symptom status will be evaluated at 1, 2, and 5 years.

**Discussion:**

This is the first prospective, multicenter, randomized, controlled study to investigate the superiority of medical therapy plus PCI over medical therapy by itself in reducing major cardiovascular events in patients presenting with coronary stenosis with “gray zone” FFR values. The results will help interventional cardiologists in making revascularization decisions regarding coronary stenosis with gray zone FFR values.

**Trial registration:**

University Hospital Medical Information Network Clinical Trials Registry, UMIN000031526. Registered on 1 March 2018.

**Electronic supplementary material:**

The online version of this article (10.1186/s13063-019-3182-1) contains supplementary material, which is available to authorized users.

## Background

Coronary physiological assessment can more effectively guide management decisions on intermediate coronary stenotic lesions than coronary anatomical assessment. This allows determination of whether the patient would benefit from revascularization or medical therapy.

Fractional flow reserve (FFR) is an established invasive index for determination of the functional severity of a coronary stenosis. FFR values < 0.75 were shown to be associated with inducible myocardial ischemia with > 99% positive predictive value [1]. The DEFER study (Deferral versus performance of percutaneous coronary intervention (PCI) of functionally non-significant coronary stenosis) has since demonstrated that PCI can be safely deferred in coronary stenosis with FFR ≥ 0.75 [2]. In addition, at 15-year follow-up, it was reported that the rate of myocardial infarction (MI) was significantly lower in the deferred group than in the revascularization group (2.2% versus 10%) [3]. On the contrary, PCI for coronary stenosis with FFR ≤ 0.80, compared with medical therapy by itself, is associated with improved clinical outcomes [4–8]. Nonetheless, revascularization for coronary stenosis with FFR between 0.75 and 0.80, the so-called gray zone, is still an unresolved issue. Owing to differing study designs and populations, previous studies have reported conflicting results regarding outcomes of revascularization versus deferral for coronary stenosis with gray zone FFR values [9–17].

We therefore will conduct a prospective, multicenter, randomized, controlled trial to investigate whether medical therapy plus PCI is superior to medical therapy alone in reducing major cardiovascular events in patients presenting with coronary stenosis with gray zone FFR values.

## Methods/design

### Study design

The Comparison of clinical outcomes following percutaneous coronary intervention versus optimal medical therapy based on gray zone fractional flow reserve in stable angina patients with intermediate coronary artery stenosis (COMFORTABLE) study is designed as a prospective, multicenter, open-label, parallel group, randomized, controlled, superiority study. This study is being conducted across 11 hospitals in Wakayama Prefecture and Tokyo, Japan. A total of 410 eligible participants will be recruited and randomized into either the performed PCI group or the deferred PCI group, with an allocation ratio of 1:1. Recruitment will begin soon.

**Fig. 1:**
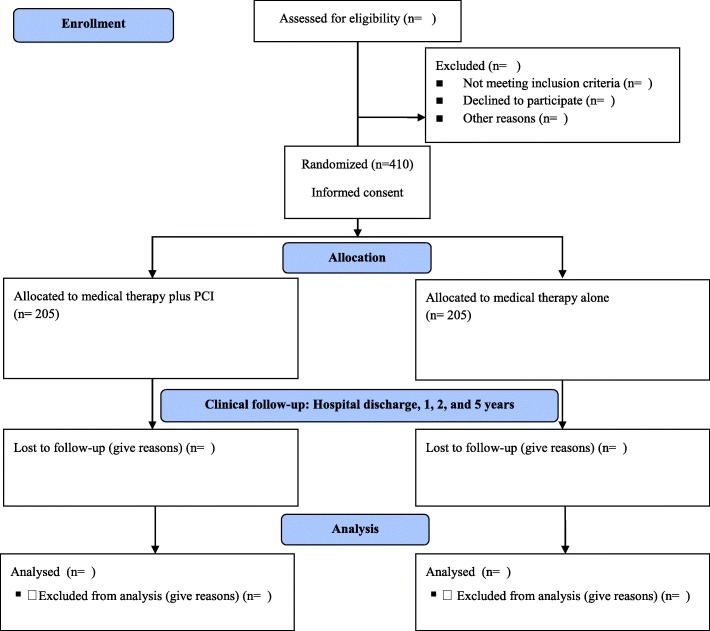
CONSORT diagram. *PCI* Percutaneous coronary intervention

**Fig. 2:**
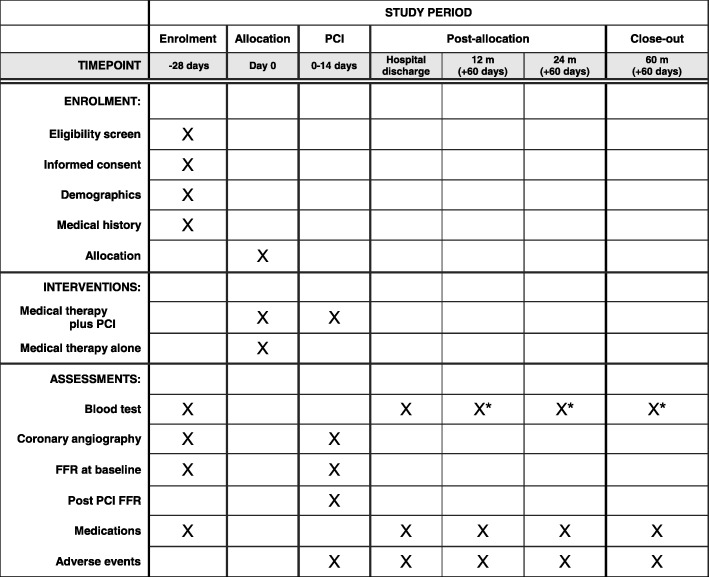
SPIRIT schedule of enrollment, intervention, and assessments. *If clinically indicated. *FFR* Fractional flow reserve, *PCI* Percutaneous coronary intervention

The protocol structure was written in compliance with the Consolidated Standards of Reporting Trials (CONSORT) 2010 statement guidelines, and it follows the Standard Protocol Items: Recommendations for Interventional Trials (SPIRIT) 2013 statement. Figure 1 shows the CONSORT diagram of this study, and Fig. 2 shows the SPIRIT schedule. The complete SPIRIT checklist for the study is provided in Additional file 1.

### Objective

The aim of this study is to determine whether medical therapy plus PCI is superior to medical therapy by itself in reducing major cardiovascular events in patients presenting coronary stenosis with “gray zone” FFR values.

### Participants

Participants will be eligible for registration in this study if they meet all of the inclusion criteria and none of the exclusion criteria listed in the subsections below.

#### Inclusion criteria

1. Intermediate coronary stenosis presenting with an FFR between 0.75 and 0.80
2. Aged ≥ 20 years
3. Able to provide written informed consent prior to any study-specific procedures

#### Exclusion criteria

1. Culprit vessel in acute coronary syndrome
2. Untreated significant lesions (percentage of diameter stenosis > 70% and/or FFR < 0.75) in coronary arteries other than the target vessel
3. Unstable hemodynamics
4. Hemodialysis
5. Previous coronary artery bypass grafting (CABG)
6. Allergy to antiplatelet therapy, anticoagulants, or contrast medium
7. Intracranial and/or gastrointestinal bleeding
8. Planned surgical procedure within 12 months
9. Life expectancy < 1 year
10. Pregnancy
11. Severe valvular heart disease
12. Participation in clinical trials of other devices or drugs
13. Left main coronary artery (LMCA) disease (percentage of diameter stenosis > 50%) or planned PCI for LMCA disease
14. Chronic total occlusions
15. Heavily calcified or tortuous vessels, meaning inability to cross the lesion with a pressure wire is expected
16. Diffuse diseases, heavily calcified lesions, small vessels, and/or aneurysmal lesions, meaning PCI is thought to be unsuitable by operators

### Randomization

Eligible participants will be allocated to one of the two groups (medical therapy plus PCI group or medical therapy alone group) by the online randomization system. Balanced randomization based on critical variables (diabetes, age, sex, and institution) will be performed automatically by the randomization algorithm. Knowledge of the treatment allocation is open to the investigators and participants.

### Sample size calculation

On the basis of event rates of previous studies that evaluated deferred versus performed revascularization in patients presenting coronary stenosis with gray zone FFR values, we predicted that the event rates at 1 year would be 6.4% in the revascularization group and 16% in the deferred group [15]. Under the above event rates and the hypothesis that the crossover rate from the medical therapy alone arm to the PCI arm is 5%, we calculated that a sample size of 368 patients (184 per arm) would provide the trial with 80% power to detect the superiority of medical therapy plus PCI over medical therapy by itself, at a two-sided alpha level of 5%. Assuming a dropout rate of 10% (patients lost to follow-up or patients unsuitable for analysis), the target sample size was set at 410 patients (205 per arm).

### Endpoints

The primary endpoint is 1-year major adverse cardiac events (MACE), defined as a combined endpoint of all-cause death, nonfatal MI, or unplanned target vessel revascularization (TVR). Secondary endpoints will include MACE at 2 and 5 years. Each individual component of the primary endpoint, cardiovascular death, target vessel-related and non-target vessel-related MI, all MI, clinically driven TVR or non-TVR, all revascularization, stent thrombosis, and angina symptom status will also be evaluated at 1-, 2-, and 5-year follow-up. All-cause death is defined as death of any cause, cardiovascular as well as noncardiovascular. MI is defined as a clinical episode of typical chest pain with development of new Q waves in at least two contiguous leads on an electrocardiogram or elevation of the creatine kinase myocardial band fraction (spontaneous, above the upper reference limit; periprocedural, more than three times the upper reference limit). Unplanned revascularization is defined as any unexpected coronary revascularization (PCI or CABG) during the follow-up period after the index procedure. TVR is defined as any repeat revascularization of any segment of the target vessel by either PCI (additional stent or angioplasty) or CABG. TVR will be considered to be ischemia-driven if revascularization is performed on a patient who has ischemic signs confirmed by noninvasive tests or FFR reassessment (with an inducible ischemia cutoff set at < 0.75), despite the presence or absence of ischemic symptoms. TVR will be considered to be clinically driven if revascularization is performed on a patient who has ischemic signs confirmed by noninvasive tests or FFR reassessment and clinical symptoms, such as chest pain that increases in frequency, intensity, or duration. Stent thrombosis is defined according to Academic Research Consortium definition [18]. Anginal status is assessed by Canadian Cardiovascular Society functional classification of angina. Clinical events are adjudicated by an independent and blinded clinical event committee.

### Coronary angiography

Coronary angiography will be performed in the standard manner via a transradial or transfemoral approach in each center. Intracoronary (IC) isosorbide dinitrate or nitroglycerin is administered in all cases preceding coronary angiography. A coronary angiogram is obtained from a standard series of six to eight projections for the left coronary artery and two or three projections for the right coronary artery. All images are stored on a CD-ROM for offline analysis. Diameter stenosis, minimum lumen diameter, lesion length, and reference lumen diameter will be measured by using dedicated software for quantitative coronary angiography (CASS-5; Pie Medical, Maastricht, The Netherlands). Quantitative coronary angiographic analysis is conducted at the Core Laboratory of Wakayama Medical University, Wakayama, Japan.

### FFR measurement

IC pressure will be measured using a pressure wire system (PressureWire, Abbot Vascular, Santa Clara, CA, USA; Smartwire, Philips Volcano, San Diego, CA, USA; Comet, Boston Scientific, Marlborough, MA, USA; or OptoWire, Zeon Medical, Tokyo, Japan). FFR measurement will be done as reported previously [17]. In brief, following pressure zero-calibration and equalization, the pressure wire will be placed in the distal part of the target coronary artery. IC nitroglycerin is administered prior to FFR measurement. FFR is calculated as the mean distal coronary pressure, measured by the pressure wire, divided by the mean aortic pressure, simultaneously measured by the guiding catheter during maximal hyperemia. Maximal hyperemia will be induced by intravenous continuous infusion of adenosine 5′-triphosphate (ATP), given at 180 μg/kg/min via the forearm or femoral vein. If maximal hyperemia is suspicious, additional IC papaverine (12 mg for left coronary artery or 8 mg for right coronary artery), IC nicorandil (2 mg for both coronary arteries), or IC ATP (50 μg for left coronary artery or 25 μg for right coronary artery) administration will be recommended. In case of contraindication to ATP, IC papaverine or IC nicorandil should be administered alternatively. After distal pressure measurement is done, the pressure sensor is pulled back to the ostium of the guiding catheter during maximal hyperemia, and both pressures are checked to exclude any transducer drift. Where drift is evident (Pd/Pa < 0.98 or > 1.02 measured at the level of the catheter tip), measurements should be repeated. Because caffeine attenuates ATP-induced hyperemia by blocking activation of adenosine A2a receptor in vascular smooth muscles, patients should refrain from consuming products containing caffeine 24 h (at least 12 h) before FFR measurement [19, 20].

### PCI

In the medical therapy plus PCI group, PCI will be performed in the standard manner according to clinical guidelines at the time of the procedure by using currently available second-generation or third-generation drug-eluting stents (DES). The choice of approach site (transradial or transfemoral) is at the operator’s discretion. Also, the type of DES and the use of intravascular imaging are at the discretion of the operator. Patients who underwent PCI will be treated with aspirin (100 mg/day) and thienopyridines (clopidogrel 75 mg/day or prasugrel 3.75 mg/day) for at least 6 months, followed by aspirin indefinitely.

### Medical therapy

Guideline-directed optimal medical therapy will be applied to all participants, regardless of the treatment arm.

### Data collection and follow-up

The data will be collected using a web-based dedicated case report form. All data management and analysis will be performed centrally at the Department of Cardiovascular Medicine at the coordinating center (Wakayama Medical University, Wakayama, Japan). Members of the coordinating Center monitor and verify the data in the participating hospitals. Clinical follow-up will be performed during hospitalization; at discharge; and at 1 year, 2 years, and 5 years after allocation. The patient’s clinical status, medications, and adverse events will be evaluated and recorded during office visits or by qualified personnel via telephone contacts if office visits are impractical or impossible.

### Safety monitoring

Safety is observed throughout the study. During the study period, all participants will be monitored and evaluated for clinical events and any other adverse events by the data and safety monitoring committee.

### Statistical analysis

Continuous variables will be summarized as mean ± SD or median (IQR) for skewed data, and comparisons will be made using an unpaired Student’s *t* test or Mann-Whitney’ *U* test as appropriate. Categorical variables will be summarized as number (%), and comparisons will be made using the chi-square test or Fisher’s exact test as appropriate. Kaplan-Meier survival analysis curves will be used to assess clinical event timelines and will be compared with the log-rank test. Also, in time-to-event analyses, the treatment groups will be compared using a Cox proportional hazards model, and the results will be expressed as an HR with 95% CI. Values of *p* < 0.05 will be considered statistically significant. All statistical analyses will be conducted using JMP Pro version 14 software (SAS Institute, Cary, NC, USA).

## Discussion

Even in the current DES era, revascularization for coronary stenosis with FFR between 0.75 and 0.80 is a matter of debate. Some studies, including our retrospective studies [12, 17], have reported that PCI compared with medical therapy alone is associated with better clinical outcomes in patients with gray zone FFR values [9, 11, 14, 15]. Conversely, other studies have shown that deferred revascularization could be the preferred initial treatment strategy for coronary stenosis with gray zone FFR values [10, 13, 16]. Thus, revascularization versus deferral decision-making for coronary stenosis presenting with gray zone FFR is controversial. However, currently available evidence is limited to registries, retrospective analyses, and single-center experiences. For FFR values between 0.75 and 0.80, the clinical decision for PCI or medical therapy has been left entirely to the operator’s discretion.

The COMFORTABLE prospective study will be the first prospective, multicenter, randomized, controlled study to prove the superiority of medical therapy plus PCI over medical therapy alone in reducing major cardiovascular events in patients presenting with coronary stenosis with gray zone FFR values. The results of the COMFORTABLE prospective study will help interventional cardiologists guide revascularization decisions for coronary stenosis with gray zone FFR values.

This study has several limitations, the most important being its open-label nature. We cannot entirely exclude the possibility that the treatment strategy will be modified, because the arm of treatment to which the patient has been allocated will be known. Second, an FFR value is limited by its reliance on the achievement of maximal hyperemia. Failure to achieve peak hyperemia, by not achieving maximal reduction in microvascular resistance, may result in overestimation of FFR and lead to wrong decision-making, especially in coronary stenosis with an FFR value in and around the “gray zone” [11]. For these reasons, in this study, we will use intravenous ATP administration to induce steady-state maximal hyperemia, and additional IC papaverine, nicorandil, or ATP should be administered if maximal hyperemia is suspicious.

## Trial status

The Wakayama Medical University Institutional Review Board approved the final version of the protocol prior to the start of the study (approval number 2234). The study is registered with the University Hospital Medical Information Network Clinical Trials Registry (UMIN000031526). The trial will open for recruitment soon.

## Additional file

Additional file 1: SPIRIT 2013 Checklist: Recommended items to address in a clinical trial protocol and related documents. (DOC 122 kb)

## Abbreviations

ATP: Adenosine 5′-triphosphate
CABG: Coronary artery bypass grafting
CONSORT: Consolidated Standards of Reporting Trials
DES: Drug-eluting stent
FFR: Fractional flow reserve
IC: Intracoronary
LMCA: Left main coronary artery
MACE: Major adverse cardiac event
MI: Myocardial infarction
PCI: Percutaneous coronary intervention
SPIRIT: Standard Protocol Items: Recommendations for Interventional Trials
TVR: Target vessel revascularization
